# Correlative Light, Electron Microscopy and Raman Spectroscopy Workflow To Detect and Observe Microplastic Interactions with Whole Jellyfish

**DOI:** 10.1021/acs.est.2c09233

**Published:** 2023-04-14

**Authors:** Jessica Caldwell, Céline Loussert-Fonta, Gaëlle Toullec, Niclas Heidelberg Lyndby, Beat Haenni, Patricia Taladriz-Blanco, Begoña Espiña, Barbara Rothen-Rutishauser, Alke Petri-Fink

**Affiliations:** † Adolphe Merkle Institute, University of Fribourg, Chemin des Verdiers 4, 1700 Fribourg, Switzerland; ‡ Laboratory for Biological Geochemistry, School of Architecture, Civil and Environmental Engineering, Ecole Polytechnique Fédérale de Lausanne (EPFL), Rte Cantonale, CH-1015 Lausanne, Switzerland; § Institute of Anatomy, University of Bern, Baltzerstrasse 2, 3012 Bern, Switzerland; ∥ Water Quality Group, International Iberian Nanotechnology Laboratory (INL), Av. Mestre Jose Veiga s/n, 4715-330 Braga, Portugal; ⊥ Department of Chemistry, University of Fribourg, Chemin du Musée 9, 1700 Fribourg, Switzerland

**Keywords:** microplastics, *Cassiopea andromeda*, medusa, correlative microscopy, Raman spectroscopy

## Abstract

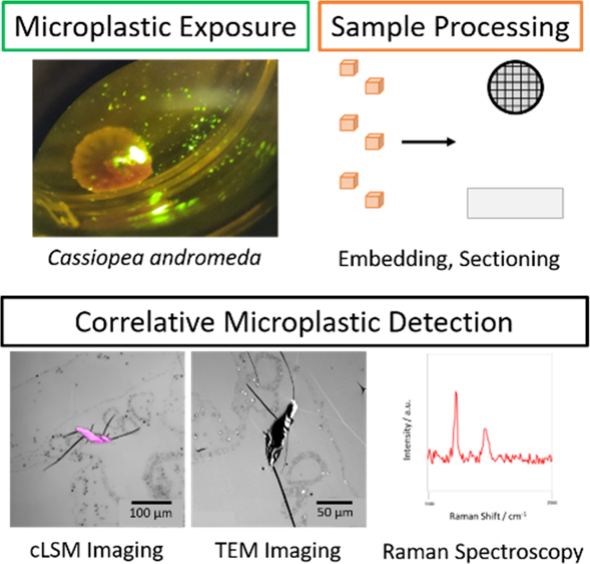

Many researchers have turned their attention to understanding microplastic interaction with marine fauna. Efforts are being made to monitor exposure pathways and concentrations and to assess the impact such interactions may have. To answer these questions, it is important to select appropriate experimental parameters and analytical protocols. This study focuses on medusae of *Cassiopea andromeda* jellyfish: a unique benthic jellyfish known to favor (sub-)tropical coastal regions which are potentially exposed to plastic waste from land-based sources. Juvenile medusae were exposed to fluorescent poly(ethylene terephthalate) and polypropylene microplastics (<300 μm), resin embedded, and sectioned before analysis with confocal laser scanning microscopy as well as transmission electron microscopy and Raman spectroscopy. Results show that the fluorescent microplastics were stable enough to be detected with the optimized analytical protocol presented and that their observed interaction with medusae occurs in a manner which is likely driven by the microplastic properties (e.g., density and hydrophobicity).

## Abstract 2

While plastic particles have been an increasingly popular research topic within the past decade, much remains uncertain about their fate within marine environments and food chains. This study presents optimized analytical protocols that allow assessment of interactions between microplastics and jellyfish to provide insight into how the properties of the plastics may influence their fate.

## Introduction

Microplastic particle (MP) (1 μm–5 mm in size) pollution of the natural environment has been highlighted as a prominent contemporary environmental concern, with scientists around the world working to gain an insight into the true magnitude and impact of the issue at hand.^1−6^ Bulk plastics are known to enter the marine environment and be degraded through photochemical and physical means into MPs, while MPs are additionally released into marine environments directly as a result of wastewater treatment plant effluent, mismanaged urban or industrial waste disposal, and rainwater runoff from roads and cities.^7−9^ Once present, these MPs are known to be rapidly dispersed into the surrounding surface water through wind and water currents and to be distributed throughout the water column and sediment as the result of intrinsic properties such as their density or external factors such as biofouling.^7,9,10^ This ubiquity has led to an increasing number of studies reporting on the interaction with and uptake of MPs by a wide variety of marine organisms, including fish, crabs, sea turtles, and even jellyfish.^10−13^

**Figure 1:**
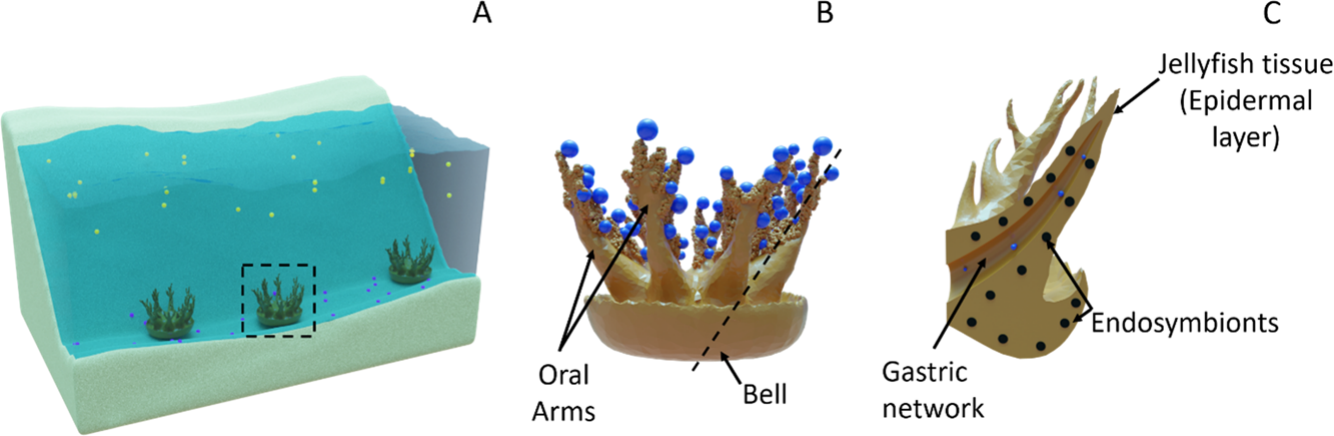
Scheme explaining the most relevant habitat and anatomy details of Cassiopea sp. (A) Representative population of medusae in a tropical coastal environment with a black dashed rectangle highlighting a single medusa for closer examination. MPs with different properties are also represented with gold (buoyant) and blue (sedimenting) spheres to aid in visualization of potential interactions. (B) Representative medusa interacting with blue MPs. Labeled arrows to indicate various relevant anatomical parts, and a dashed black line to indicate the region displayed in (C). (C) Cross-section view of a single oral arm labeled with arrows to indicate relevant anatomical parts and the photosynthetic endosymbiotic algae known to be present within the jellyfish.^22^ Generally, food and other substances from their surrounding environment will be trapped by the oral arms of Cassiopea sp. and can then be ingested through their secondary mouths and transferred to the main stomach for digestion.

While once regarded as playing a relatively inconsequential role within marine food chains, more recent literature has demonstrated that jellyfish are prey for everything from sea turtles to birds, which makes them an important new potential pathway for trophic transfer of marine contaminants.^14−17^*Cassiopea* species (sp.) are benthic jellyfish (Figure 1) that inhabit the sea floor in shallow, (sub-)tropical coastal waters. Their close proximity to land makes them interesting for study because they are potentially exposed to high initial concentrations of land-based MPs released into the marine environment.^11,15,17^ This is particularly promising when considered in tandem with their reported ability to retain pollutants over the course of a few days to weeks, providing researchers with an opportunity to study short-term fluctuations in MP levels within a region.^11,15,17^ These characteristics as well as others (e.g., the potential to stir up sedimented MPs due to their benthic nature, their ease of collection, and ease of cultivating in a laboratory) serve to support the idea that *Cassiopea* sp. jellyfish could function as bioindicator species for pollutants such as MPs.^18−21^

Despite such promising qualities, few studies exist to date which focus on the interaction of MPs with jellyfish, and the reported results often differ.^23,24^ As an example, Sucharitakul et al. and Costa et al. both studied the impact of MP exposure on *Aurelia* sp. jellyfish. Within their study, Sucharitakul et al. found that there was limited ingestion and no hazardous effects after exposure to polystyrene (PS) MPs, while Costa et al. reported ingestion and negative physiological impacts after exposure to polyethylene (PE) MPs.^23,24^ Still fewer studies have focused on the interaction of MPs with *Cassiopea* sp. jellyfish specifically. Iliff et al. demonstrated that some MPs were present in their wild-caught *Cassiopea* sp. samples, but as they were pre-processed with chemical digestion of the jellyfish tissue to make detection easier, all potential spatial information for the MPs within the tissue was lost.^11^

This highlights the compromises which must be made due to the multiple analytical challenges presented by MPs. Such challenges are the result of limitations for common techniques that are influenced by factors such as the MP’s small size, low estimated environmental concentration, and organic composition, as well as the high variability in biotic and abiotic factors within the surrounding natural environments of interest.^3,25^ Thus, it is clear that there is a need in the field for optimization of analytical techniques that will allow for the detection of MPs within marine organisms. To that end, juvenile medusae of the jellyfish *Cassiopea* sp., specifically, *Cassiopea andromeda* (*C. andromeda*), were exposed to thermostable, fluorescently labeled poly(ethylene terephthalate) (PET) and polypropylene (PP) MPs (<300 μm). Samples of *C. andromeda* medusae with MPs were then resin embedded, sectioned, and analyzed with confocal laser scanning microscopy (cLSM), transmission electron microscopy (TEM), and Raman spectroscopy to detect and study any interactions occurring (Scheme S1).

While correlative microscopy with TEM providing a high-resolution validation of initially observed fluorescence signals has been highly utilized in other fields, very little work has been conducted to optimize this work flow for MP detection in tandem with Raman spectroscopy in whole marine organisms.^26,27^ Additionally, it is often not possible to image the fluorescence of a sample after the necessary pre-processing steps for TEM due to the degradation of the molecules which give fluorescence (e.g., thermal degradation, pH degradation, or potential photobleaching during resin permeation and curing, and/or binding of heavy metal stains needed to improve TEM contrast) or to collect Raman spectra from highly fluorescent bulk samples without sacrificing signal intensity through the use of lower energy near infrared lasers.^28−31^ Thus, our work focused on overcoming these analytical concerns for the first time for samples of whole marine organisms exposed to MPs. A protocol was developed which allowed for an initial insight into how interactions with *C. andromeda* medusa may differ depending on the MP type, providing a platform for future research which can further probe such differences in these and other marine organisms.

## Methods

### MP Creation

The exact protocol for creation and characterization of the MPs utilized in this study has been previously described by Caldwell et al.^32,33^ Briefly, stock pellets of PET were purchased from Goodfellow Cambridge Ltd., and pellets of isotactic PP were purchased from Sigma-Aldrich. These pellets underwent a sequential melt processing and milling protocol to create the final MP stocks used for the study. First, thin films of the as-received pellets were prepared by compression molding 2.5 g of each polymer between Kapton sheets in a hot press (Carver) at 170 °C for PP or 255 °C for PET at 2 tons for 1 min and then 5 tons for an additional 1 min. The films were removed from the press and cooled to room temperature. To create fluorescently labeled plastics, the dye 1,4-bis(α-cyano-4-methoxystyryl)-2,5-dimethoxybenzene (C1RG) was added onto the films in concentrations of 0.01% (by weight) for PP (PPC1RG) and 0.1% for PET (PETC1RG). The films with dye were folded to seal in the dye and compression-molded again using the parameters previously described. The films of labeled or unlabeled plastic were fed into a Haake Mini Lab II twin screw extruder (Thermo Fisher) for two 5 min mixing cycles under recycling conditions at 170 °C (PP) or 255 °C (PET) to ensure even mixing and obtain extruded filaments of plastic which could be cut into pellets. These pellets were placed in a polycarbonate chamber with a steel milling rod and steel chamber plugs to be milled under cryogenic conditions in a 6770 Freezer Mill (SPEX) with a 15 min pre-cooling followed by 2 cycles of 3 min milling at 12 cycles per second (cps) with a 2 min cooling period in between. Finally, the cryo-milled MPs were sieved with a 0.3 mm stainless-steel mesh (VWR International) to select for the size range of interest.

### Scanning Electron Microscopy (SEM)

To assess their size and shape, MPs were placed onto aluminum SEM holders (Agar Scientific) and 10 μL of ethanol (VWR Chemicals) was dropped onto the sample to disperse them across the surface of the holder. Samples were dried overnight prior to sputter coating a 2.5 nm layer of gold using a 208 HR sputter coater (Cressington Scientific Instruments). All imaging was carried out with a Mira3 LM FE scanning electron microscope (pixel size: 0.0017 × 0.0017 μm^2^; Tescan).

### *C. andromeda* Cultivation

*C. andromeda* juvenile medusae with bell diameters of 3–5 mm were kept in 150 L of artificial sea water with a temperature of 26 °C, a pH of 8–8.2, and a salinity of 39 ppt. A 12:12 day:night cycle was maintained with an average downwelling irradiance of 150 μmol photons m^–2^ s^–1^ using cold, white LED light. Feeding was conducted two to five times a week with *Artemia* nauplii (ephyra to sub-adults four to five times, or adults two times). To maintain clean tank conditions, UV, mesh (200 μm), and skimmer filtration were utilized, and the tank was cleaned twice a month with a 30 L water renewal.

### Experimental Setup for Exposures

An aliquot of water from the main *C. andromeda* tank was collected and passed through a 0.22 μm syringe filter (Macherey-Nagel) to remove large organic matter. Subsequently, 1 mL per well of filtered tank water was pipetted into 16 wells in two different 12-well plates. A transfer pipette (Merck) was cut and used to transfer a single *C. andromeda* medusa at a time into the wells of 12-well plates. MPs, which had been pre-massed using an AG204 Delta Range balance (Mettler-Toledo) into labeled Eppendorf tubes (Sigma-Aldrich) to ensure a final exposure concentration of 120 μg/mL, were suspended in 200 μL of filtered tank water, mixed thoroughly, and then pipetted into the appropriately labeled well with the medusa (Table S2). Each tube was rinsed with additional 300 μL of filtered tank water to ensure that all MPs were removed. The rinse water was added to the respective well so that the final volume of water introduced with plastic MPs was 500 μL. For the blank control samples, 500 μL of filtered tank water without plastic particles was used. An additional 1 mL of filtered tank water was introduced per well to help facilitate mixing. A small aliquot of water was taken from an *Artemia* hatcher and filtered, and then 20 μL of filtered *Artemia* hatcher water was dropped into each well to facilitate feeding behavior. The 12-well plates were placed in an incubator to ensure a constant temperature of 25 °C and light exposure of 150 μmol photons m^–2^ s^–1^ per organism during a 6 h exposure period.

### Fixation and Sectioning

At the end of the 6 h exposure, transfer pipettes were used to move each juvenile medusa to a clean Eppendorf tube and 1.5 mL of a fixative solution (pH 8) containing 9% (by volume) sucrose (Sigma-Aldrich), 4% paraformaldehyde (Electron Microscopy Sciences), and 2.5% glutaraldehyde (Electron Microscopy Sciences) was added. All water was removed, and samples were maintained in fixative at room temperature 2 h before storing at 4 °C until further work was conducted.

Sample dehydration with a graded series of ethanol solutions (30% ethanol 30 min, 50% ethanol 30 min, 70% ethanol 15 min, 90% ethanol 30 min, and then three times 100% ethanol 30 min each) was conducted prior to an infiltration step with a graded series of EPON resin:100% ethanol mixtures (1:3 1 h, 1:1 1 h, 3:1 1 h), two times in pure resin 1 h each, and then a final resin bath with catalyzer for 12 h. Samples were then left to polymerize for 3 days at 60 °C.

Fully polymerized resin blocks could be trimmed, and micron-sized step sections could be made until a viable start position for analysis was reached using a Leica UC6 Ultramicrotome (Leica). Once the block was adequately trimmed, serial ultrathin sections of 400 nm thickness were cut and placed onto glass microscopy slides (Thermo Fisher Scientific). Subsequent ultrathin sections of 70 nm thickness were then cut and placed onto carbon film on copper 300 square mesh grids (Electron Microscopy Sciences).

### Sample Staining

To improve contrast for bright field imaging, all sample sections on glass microscopy slides were stained with a toluidine blue solution (Merck). All slides containing sample sections were placed, samples facing up, on a hot plate at 50 °C for 2 min prior to coating the full slide surface with a 0.5% toluidine blue solution. After 3 min, the slides were removed from the hot plate and rinsed copiously with Milli-Q water to remove excess staining solution. Slides were returned to the hot plate until dry.

To improve contrast for TEM imaging, sample sections on TEM grids were stained with commercially available UranyLess (Electron Microscopy Sciences) and lead citrate (Electron Microscopy Sciences) solutions in a protocol adapted from the manufacturer recommended use. Per sample grid, a single drop of UranyLess was prepared in addition to three wash droplets of 100 μL of Milli-Q water followed by a single drop of lead citrate and three final 100 μL droplets of Milli-Q water. Grids were placed for 1 min into the UranyLess, removed, blotted with a Kim wipe, and then placed onto the first Milli-Q droplet for 5 min. This wash process was repeated twice for a total of three Milli-Q wash steps. After the third wash, samples were blotted and transferred to a drop of a lead citrate for 5 min. The grids were removed from lead citrate, blotted, and then placed onto the first 100 μL Milli-Q wash droplet for 5 min. This wash process was repeated twice for a total of three Milli-Q wash steps. Finally, the grids were removed from the last Milli-Q wash, blotted, and left to dry overnight at room temperature.

### Confocal Laser Scanning Microscopy (cLSM)

All fixed samples were imaged with a Zeiss LSM 710 META confocal microscope (Carl-Zeiss AG). Excitation laser wavelengths of 488 nm (C1RG) and 440 nm (Chlorophyll) were utilized for fixed samples prior to resin embedding. Resin embedded samples were imaged with bright field microscopy parameters and 488 nm (C1RG) excitation.

### Transmission Electron Microscopy (TEM)

All TEM samples were imaged with a Tecnai Spirit transmission electron microscope (FEI) operating at 120 kV with a wide angle Veleta CCD camera (2048 × 2048 pixel; Olympus).

### Raman Spectroscopy

All Raman measurements were conducted with a WITec Alpha300 Access confocal Raman microscope using a 633 nm laser with a power of 1–5 mW, 20× or 50× magnification air objectives, and a built-in CCD camera for obtaining the bright field images (WITec). TEM grids and loose MP powders were supported on clean glass coverslips to ensure that the samples were not lost. Raw spectral data were extracted from the accompanying WITec Control 5 software so that cosmic ray removal (zap) and baseline corrections (multi-point baseline subtraction) could be conducted using Grams AI software (version 9.3; ThermoFisher Scientific). The final presented spectra were obtained by accumulating multiple (300–1000) 0.5 s measurements and averaging them. A full table of the exact measurement parameters for each of the presented spectra can be seen in Table S3.

### Data Processing

Tile scan stitching was conducted automatically through the accompanying Zen 2010 software of the Zeiss cLSM. All additional image processing for SEM, cLSM, and TEM, including scale bar inclusion, channel merging, look up table application, contrast adjustments, and noise reduction, was conducted using Fiji (ImageJ version 1.53).

### Contamination Prevention and Controls

During exposure experiments, covered sample plates were used to limit the risk of atmospheric deposition of contaminants. Multiple control exposures were conducted, ensuring a control was present in each sample plate used, and the blank samples were processed for imaging in an identical manner to true samples. Images were acquired for “empty” control regions (e.g., only ethanol dropped and sputtered on an SEM stub, empty resin regions). Furthermore, slides were stored in closed containers during all procedural steps which did not directly involve their handling and during the time between sample creation and analysis. When samples were handled, cotton lab coats and latex gloves were worn.

## Results

### MP Characterization

Representative sub-samples for each of the MP stocks imaged with SEM (Figure S1) showed high heterogeneity in the shape, size, and surface roughness of the particles present. Measurements obtained from these images indicated that all MPs were, at their largest point, 300 μm or less in size (Table S1) and thus smaller than the average size of newly hatched *Artemia* nauplii (e.g., ∼0.4 mm) *C. andromeda* were fed, but within a size range comparable to that of MPs reported to be found in wild-caught jellyfish.^34,35^ Procedural blank images (Figure S2) obtained under the same conditions showed no dust or other particulate matter contamination within the relevant size range.

As an initial method validation and control, 1,4-bis(α-cyano-4-methoxystyryl)-2,5-dimethoxybenzene (C1RG) labeled MPs were embedded into EPON epoxy resin and sectioned for cLSM imaging (see Methods section for more details). Representative images of the MP stocks in resin can be seen in Figure S3 with the fluorescence clearly visible for both PPC1RG MP and PETC1RG MP samples sectioned at varying thicknesses. This combination of SEM sizing and cLSM imaging sectioned C1RG labeled MPs confirmed that the MP stocks were viable for further use. Thus, each MP type was prepared via mass balancing to obtain samples whose exposure concentration would be 120 μg/mL. Due to physical limitations based on the MP properties and experimental conditions (e.g., sample preparation by mass balancing with buoyant or sedimenting particles resulting in no possibility to evenly dilute a stock to lower concentrations), the concentrations it was possible to prepare were higher than anticipated environmental contamination levels of a few micrograms to nanograms per liter.^25^

### MP Detection Prior to Resin Embedding

**Figure 2:**
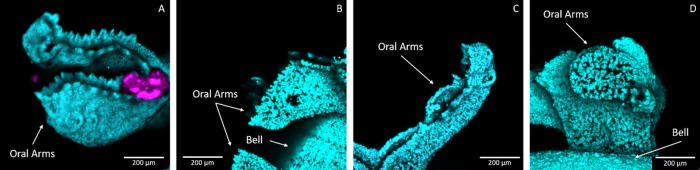
Z-projections of maximum fluorescence intensity values for fixed medusae after the 6 h exposure period. The fluorescence of C1RG labeled plastic particles was imaged with EGFP excitation/emission filters and is shown in magenta while the chlorophyll excitation/emission in cyan allowed for imaging of the endosymbiont within the *C. andromeda* tissue. (A) PETC1RG MP exposed medusa with MP immobilized near a secondary mouth on an oral arm. (B) Oral arm and portion of the bell of an unlabeled PET MP exposed medusa. (C) Oral arm of a PPC1RG MP exposed medusa. (D) Multiple oral arms and a portion of the bell of an unlabeled PP MP exposed medusa.

Upon chemical fixation of the juvenile *C. andromeda* medusae exposed to 120 μg/mL of MPs for 6 h, cLSM imaging was conducted to probe whether the MPs had interacted with the medusae and select the sample regions of interest (ROIs) for further processing. Representative cLSM Z-projections shown in Figure 2 allowed for spatial detection of C1RG fluorescence in the oral arms of a medusa exposed to PETC1RG MPs (Figure 2A; PETC1RG fluorescence in magenta). Specifically, the PETC1RG MP shown in Figure 2A appears to be physically trapped within the fringed digitate surrounding the secondary mouths of the oral arms, indicating that the MP was in the initial stages of being ingested. Oral arms of medusae exposed to labeled or unlabeled PP MPs as well as the medusa exposed to unlabeled PET MPs did not have any detectable C1RG fluorescence. This result is expected for unlabeled MP samples, but for the PPC1RG MP exposed medusa, a lack of fluorescence indicates that there are no MPs present. Due to the size of the whole organisms (bell diameters of 3–5 mm) in comparison to the viable working distance (0.55 mm) for the microscope objective utilized, it was not possible to completely rule out the presence of MPs within the relevant samples without further sample processing, highlighting the need for the resin embedding and sectioning protocol which was optimized using the MP stocks.

### MP Detection in Final Sample Sections

**Figure 3:**
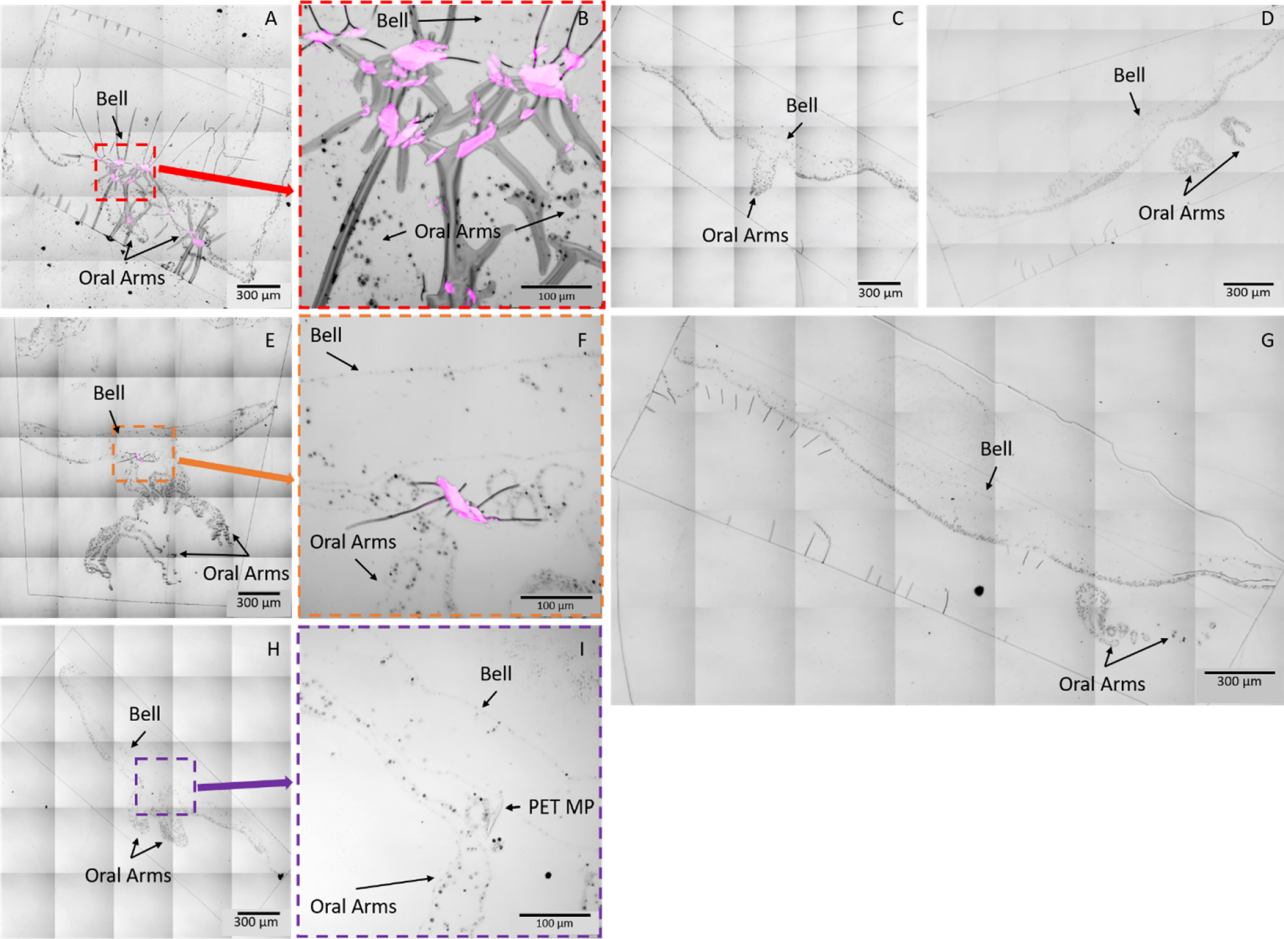
Tile scans of the final 400 nm thick resin-embedded sample sections placed on glass slides. The fluorescence of C1RG labeled plastic particles was imaged with EGFP excitation/emission filters and is shown in magenta while the bright field imaging was used to visualize toluidine blue stained *C. andromeda* epidermal tissue and endosymbionts. Samples which do not contain plastic particles do not have ROIs shown in additional images, but those which were observed to contain MPs have ROI images presented for a closer look at the MPs. (A) PETC1RG MP exposed medusa 1 with a dashed red rectangle and red arrow indicating the region of interest presented in (B), (C) PPC1RG MP exposed medusa, (D) unlabeled PP MP exposed medusa, (E) PETC1RG MP exposed medusa 2 with the orange dashed rectangle and orange arrow indicating the region of interest presented in (F), (G) negative control medusa which was not exposed to MPs, and (H) unlabeled PET MP exposed medusa with the purple dashed rectangle and purple arrow indicating the region of interest presented in (I).

cLSM imaging of the final resin embedded sample sections on glass microscopy slides (Figure 3) confirmed the presence of PETC1RG MPs in two of the three exposed medusae (henceforth referred to as PETC1RG exposed medusa 1 and 2, respectively) as well as unlabeled PET MPs in one of the three exposed medusae. The MPs observed in these images are present within the gastric cavities, indicating that the PET and PETC1RG MPs were ingested. All PP exposed medusae as well as the control medusae do not show the presence of any MPs. Imaging multiple sequential sections ensures that the initial presence or absence of MPs observed was not the result of artifacts, as well as allowing tracking of MP position through the whole organism where relevant (Figures S4–S8).

**Figure 4:**
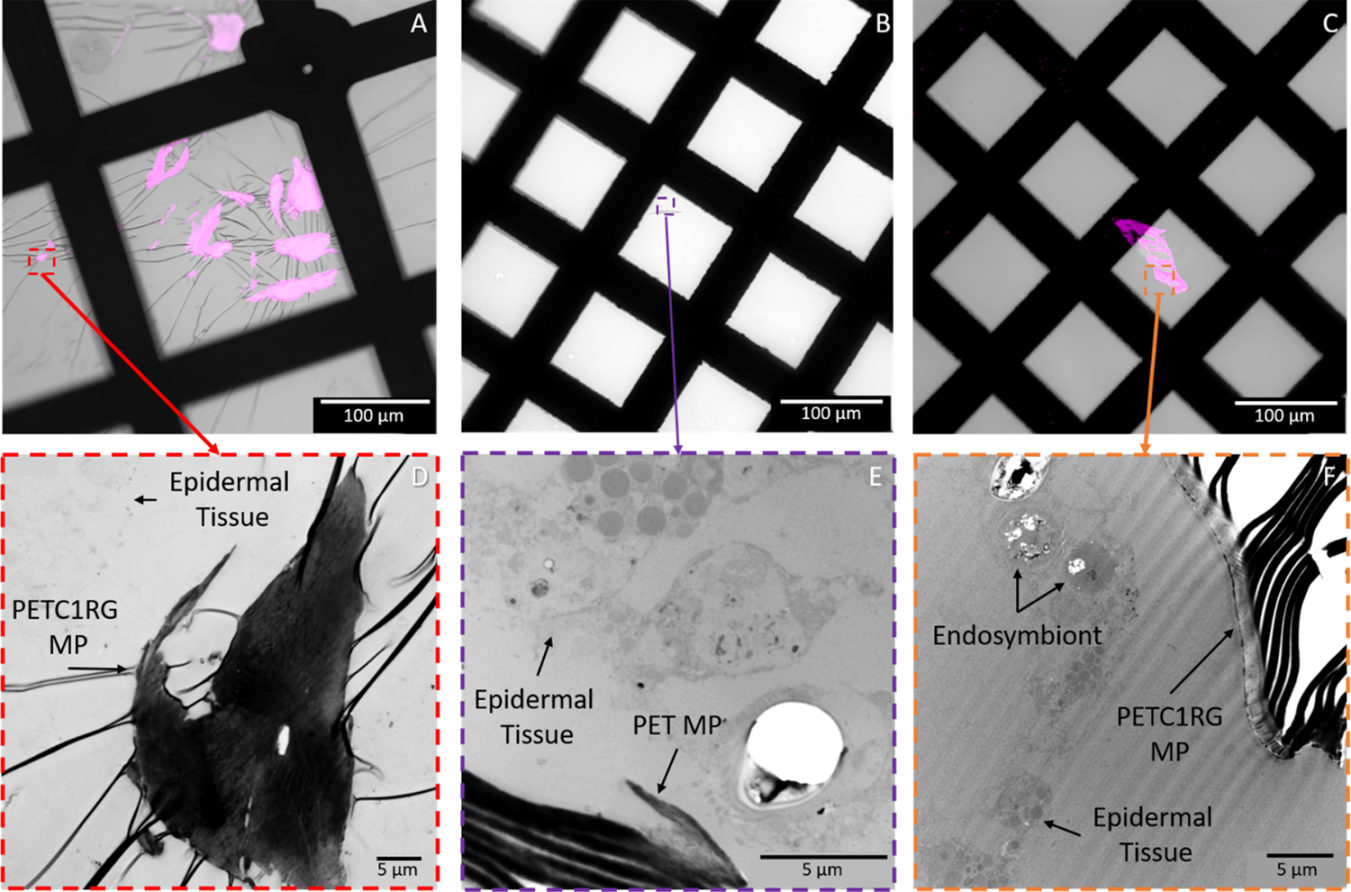
cLSM and TEM images from the final 70 nm thick resin-embedded sample sections placed on TEM grids. This figure shows only samples which were found to contain plastic particles. In the cLSM, the fluorescence of C1RG labeled plastic particles was imaged with EGFP excitation/emission filters and is shown in magenta while the bright field imaging was used to visualize toluidine blue stained *C. andromeda* epidermal tissue and endosymbionts as well as the TEM grid itself. (A) cLSM image of PETC1RG MP exposed medusa 1 with a red dashed rectangle and red arrow highlighting the region of interest shown in the TEM image in (D), (B) cLSM image of unlabeled PET MP exposed medusa with a purple dashed rectangle and purple arrow highlighting the region of interest shown in the TEM image in (E), and (C) cLSM image of PETC1RG MP exposed medusa 2 with an orange dashed rectangle and orange arrow highlighting the region of interest shown in the TEM image in (F). Representative examples of key features of interest in TEM images have been labeled with arrows for clarity.

Tile scan imaging of the full sample sections placed on TEM grids allows for identification of specific landmarks (i.e., MPs, resin edges, and grid center) which were important for location triangulation in subsequent TEM imaging (Figure S9). For PETC1RG samples, the C1RG fluorescence could be clearly observed even with sample sections of only 70 nm thickness. However, it is apparent that gaps are present in some MPs observed either as the result of uneven surface topography in the MPs themselves or of slight sample damage during sectioning. Despite this, correlative imaging with TEM was possible and the exact ROIs imaged in cLSM could be observed at significantly higher magnification (Figures 4 and S10). Correlation with electron microscopy allows for further validation of the MP presence, which is of particular importance for the unlabeled MPs, and serves to confirm that the MPs are located inside the gastric cavities.

**Figure 5:**
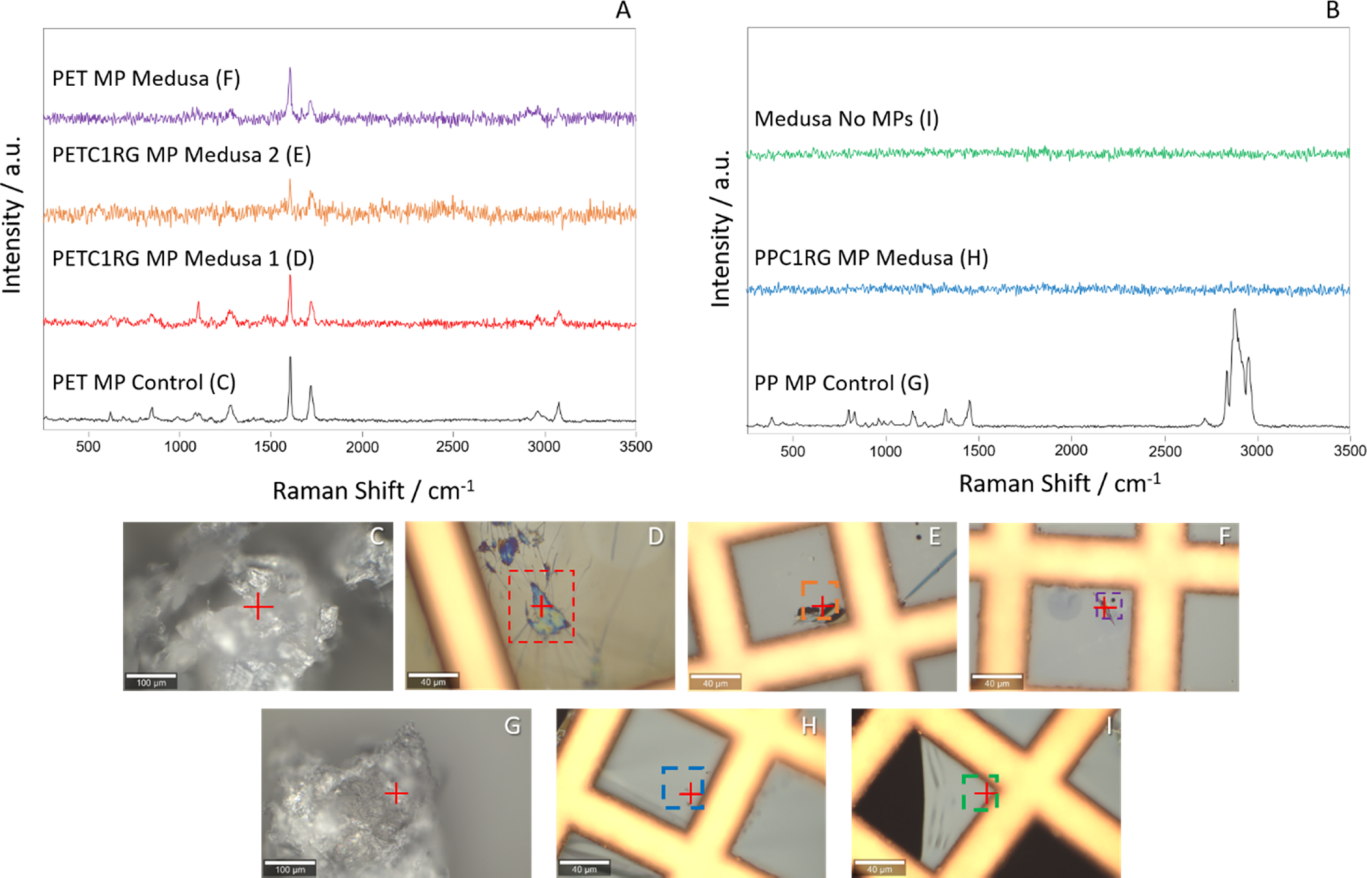
Raman spectra measured for sectioned TEM samples and MP controls shown above their bright field ROI images. Exact measurement positions are indicated with red crosses in the images, and the scale bar represents 40 μm for TEM sections and 100 μm for the MP controls. (A) Spectrum for the PET MP control measured on glass set beneath all spectra collected for medusae exposed to PETC1RG and PET MPs. The figure letter of the relevant ROI image for each spectrum is listed in parentheses next to the spectrum label. (B) Spectrum for the PP MP control beneath the spectrum for the medusa exposed to PPC1RG MPs and the control medusa not exposed to MPs. The figure letter of the relevant ROI image for each spectrum is listed in parentheses next to the spectrum label. (C) ROI image for the PET MP control spectra. (D) ROI image for the PETC1RG MP exposed medusa 1 with a red dashed box to indicate the region which overlaps with TEM images. (E) ROI image for the PETC1RG MP exposed medusa 2 with an orange dashed box to indicate the region which overlaps with TEM images. (F) ROI image for the PET MP exposed medusa with a purple dashed box to indicate the region which overlaps with TEM images. (G) ROI image for the PP MP spectra. (H) ROI image for the PPC1RG exposed medusa with a blue dashed box used to indicate correlation with TEM images. (I) ROI image for the blank control medusa with a blue dashed box used to indicate correlation with TEM images. The stacked Raman spectra do not have the same *Y*-axis scale and thus provide qualitative observations of the chemical fingerprint.

As a final validation, material-specific detection of MPs could be conducted through chemical fingerprint acquisition with a confocal Raman microscope for the ROIs previously imaged. For PET and PETC1RG exposed medusae, spectra could be obtained in regions where particles were observed in the TEM. Within these regions, it was possible to detect peaks that have been reported in the literature to be indicative of PET presence, including the two primary peaks of interest at ∼1615 to 1620 cm^–1^ due to aromatic ring bending vibrations and ∼1730 cm^–1^ due to the carbonyl stretching mode (Figure 5).^36,37^ Comparing these measurements to PET MPs which are not embedded in resin shows good agreement, further confirming the presence of PET within the medusa sample sections (Figure 5). However, the PP and PPC1RG exposed samples did not show any signal in the primary region of interest known to be indicative of PP (e.g., the −CH_2_ and −CH_3_ deformation vibrations from 2600 to 3000 cm^–1^), and showed none of the peaks observed in the MP control measurement (Figure 5).^38^ Additional measurements of regions within the sample sections for the medusa not exposed to MPs also reveal no peaks in any of the relevant regions, confirming that the signal observed for PET and PETC1RG comes from the MPs themselves (Figure 5).

## Discussion

Initial characterization of the MPs used in this study revealed that the top-down mechanical degradation protocol used to create them resulted in a high heterogeneity in the size, shape, and surface roughness of the MP stocks. This heterogeneity makes the MP stocks a good representation of MP fragments commonly reported to be collected in environmental sampling campaigns.^7^ Furthermore, PET and PP represent two distinct types of MP that are commonly found in environmental campaigns and are interesting for comparative purposes due to differences in relevant properties such as density and hydrophobicity.^3,7^ PP is less dense (0.90 g/cm^3^) than water and PET (1.35 g/cm^3^). Furthermore, PP has been previously shown to be more hydrophobic than PET (Table S1).^32^ These properties directly impact the behavior of the MPs during the exposure experiments, with PET and PETC1RG MPs sedimenting over time while PP and PPC1RG MPs were buoyant and only mixed into lower parts of the water column as a result of currents in the water created by the movement of the *C. andromeda* medusa. As *C. andromeda* are benthic by nature, they remain at the bottom of the well (or tank) which they are in. Thus, imaging revealed that the differences in properties of the MPs could already be observed to impact their interactions with *C. andromeda*. PET and PETC1RG exposed medusae were shown to grab the MPs with their oral arms and ingest them, while no such interaction was observed for PP and PPC1RG exposed medusae. Findings reported by Iliff et al. and Sun et al. also showed that polyesters (e.g., PET) were among the most common MP type observed in wild-caught jellyfish samples.^11,35^ This highlights an interesting potential for future studies which focus on further probing such differences through altering experimental conditions such as exposure time, probing whether these differences in interaction remain after the MPs are biofouled (i.e., covered with various organic materials from natural sources), and conducting MP exposures to *Cassiopea* sp. at different stages in the life cycle. Furthermore, work could be conducted to expose the *Artemia* sp. prey to MPs prior to *Cassiopea* sp. feeding in order to gain insight into potential biomagnification of MPs across multiple trophic levels as was recently proposed for PP MPs by Jeyavani et al. or observed for PS MPs with *Aurelia coerulea* by Sucharitakul et al.^39,40^ As a final consideration, researchers could attempt to enact a more complex exposure setup which better mimics the anticipated physical environment (e.g., simulating water currents and waves for more accurate mixing conditions). Such studies would allow researchers to gain further insight into why certain MP types appear to be preferentially ingested and what factors influence the interactions which occur.

In addition to confirming the relevant physical properties were as desired, it was possible to confirm that the C1RG fluorophore utilized to create the labeled MP stocks was not degraded by the resin embedding process and could still be observed even after ultrathin sectioning and heavy metal staining (UranyLess and lead citrate). While this was already beneficial for observing the MP locations on the TEM grids to facilitate ROI selection (Figure S9), such a property would be of greater importance for smaller plastic particles such as submicron (100 nm–1 μm) or nanoplastics (<100 nm; European Union definition of “nano”) which cannot be detected in regular bright field microscopy due to resolution limits and contrast issues.^3,41^ This provides the potential for future studies to sequentially mill plastic particles to smaller sizes and study their interactions with organisms when their movement in water would be driven by Brownian motion as opposed to buoyancy or sedimentation caused by the plastic’s density.^32,42^

The analysis of samples exposed to plastic particles at sizes below the resolution limit for optical microscopies (i.e., <200 nm as reported for Rayleigh diffraction limited resolution) would be further supported by the high resolution one can obtain with a TEM.^43−45^ In this study, TEM imaging was already shown to be valuable for assessing exact MP location as it was possible to image the MPs along with the cells of the epidermal layer and endosymbionts of *C. andromeda* medusae. It was further possible to validate that suspected particles observed in the gastric cavity of the medusa exposed to unlabeled PET MPs were true solid materials and not some sort of resin defect. This confirmed that the MPs were present within the sample sections and stand in good agreement with findings reported by researchers such as Costa et al., who observed that imaging with confocal microscopy alone was not necessarily sufficient to confirm the presence of plastic particles in the *Aurelia* sp. jellyfish they studied.^23^

Further investigation into the exact properties of the particles being imaged through the use of Raman spectroscopy, a technique which yields spectra based on material-specific inelastic scattering of light, serves as the final validation that of MP presence or absence within the sample of interest. These measurements could be conducted with a 633 nm excitation laser and multiple accumulations as the thin sample section could be photobleached more readily than a bulk sample. However, this prolonged photobleaching is not necessary for samples which are not fluorescently labeled. Regardless of if the MPs were fluorescently labeled, Raman spectra could be obtained for all sections observed to contain PET and PETC1RG MPs, indicating that the protocol utilized is reliably reproducible. The lack of Raman peaks for the PPC1RG MP exposed medusa and the medusa not exposed to MPs is in good agreement with the findings from the cLSM and TEM, where no MPs could be observed. Thus, Raman spectra serve to confirm that in this experiment, the PET and PETC1RG MPs were ingested by the *C. andromeda* medusae, while the PP and PPC1RG MPs were not.

Such findings demonstrate that with the work flow presented in this study (Scheme S1), one would be able to analyze not just samples with labeled reference MPs, but also true environmental samples as well. Thus, the protocol could be employed as a complimentary analysis for studies such as the one conducted by Iliff et al. where *Cassiopea* sp. jellyfish were collected from their natural habitat to check for interaction with MPs. In such an analysis, one could select a few representative jellyfish for sectioning, imaging, and spectroscopy, then digest the remaining jellyfish, and present average MP per organism count data alongside representative images of where MPs are located within the organisms. This would allow for a more holistic understanding of MP interaction with *Cassiopea* sp. in their native environment and set the foundation for utilizing studies of MP presence in *Cassiopea* sp. jellyfish to monitor the overall pollution levels in the region they are collected from.

## Data Availability

The raw data presented in this manuscript have been uploaded in Zenodo under the DOI 10.5281/zenodo.7780575.

## Supporting Information Available

A document containing a schematic overview of the sample preparation pathway, additional microscopy data (TEM, SEM, and cLSM), and tables listing particle characterization data and experimental parameters for MP exposures and Raman spectroscopy (PDF)
