# Supplementary material for: Correlative Light, Electron Microscopy and Raman Spectroscopy Workflow To Detect and Observe Microplastic Interactions with Whole Jellyfish
Source: Environ Sci Technol. 2023 Apr 14;57(16):6664–72. doi: 10.1021/acs.est.2c09233 (PMC10134485; doi:10.1021/acs.est.2c09233)
Supplement: Supplementary file 1 — es2c09233_si_001.pdf [file es2c09233_si_001.pdf]

Supplementary Information: Correlative Light, Electron Microscopy and Raman Spectroscopy Workflow to Detect and Observe Microplastic Interactions with Whole Jellyfish

Jessica Caldwell[1,], Céline Loussert-Fonta[1], Gaëlle Toullec[2], Niclas Heidelberg Lyndby[2], Beat Haenni[3], Patricia Taladriz-Blanco[4], Begoña Espiña[4], Barbara Rothen-Rutishauser[1], Alke Petri-Fink[1,5,*]*

[1]Adolphe Merkle Institute, University of Fribourg, Chemin des Verdiers 4, 1700 Fribourg, Switzerland

[2]Laboratory for Biological Geochemistry, School of Architecture, Civil and Environmental Engineering, Ecole Polytechnique Fédérale de Lausanne (EPFL), Rte Cantonale, CH-1015 Lausanne, Switzerland

[3]Institute of Anatomy, University of Bern, Baltzerstrasse 2, 3012 Bern, Switzerland

[4]Water Quality Group, International Iberian Nanotechnology Laboratory (INL), Av. Mestre Jose Veiga s/n, 4715-330, Braga, Portugal

[5]Department of Chemistry, University of Fribourg, Chemin du Musée 9, 1700 Fribourg, Switzerland

**Jessica Caldwell: jessica.caldwell@unifr.ch / jessic203@yahoo.com; Alke Petri-Fink: alke.fink@unifr.ch*

CONTENTS

- Schemes: 1
- Figures: 10
- Tables: 3
- Total Pages: 13

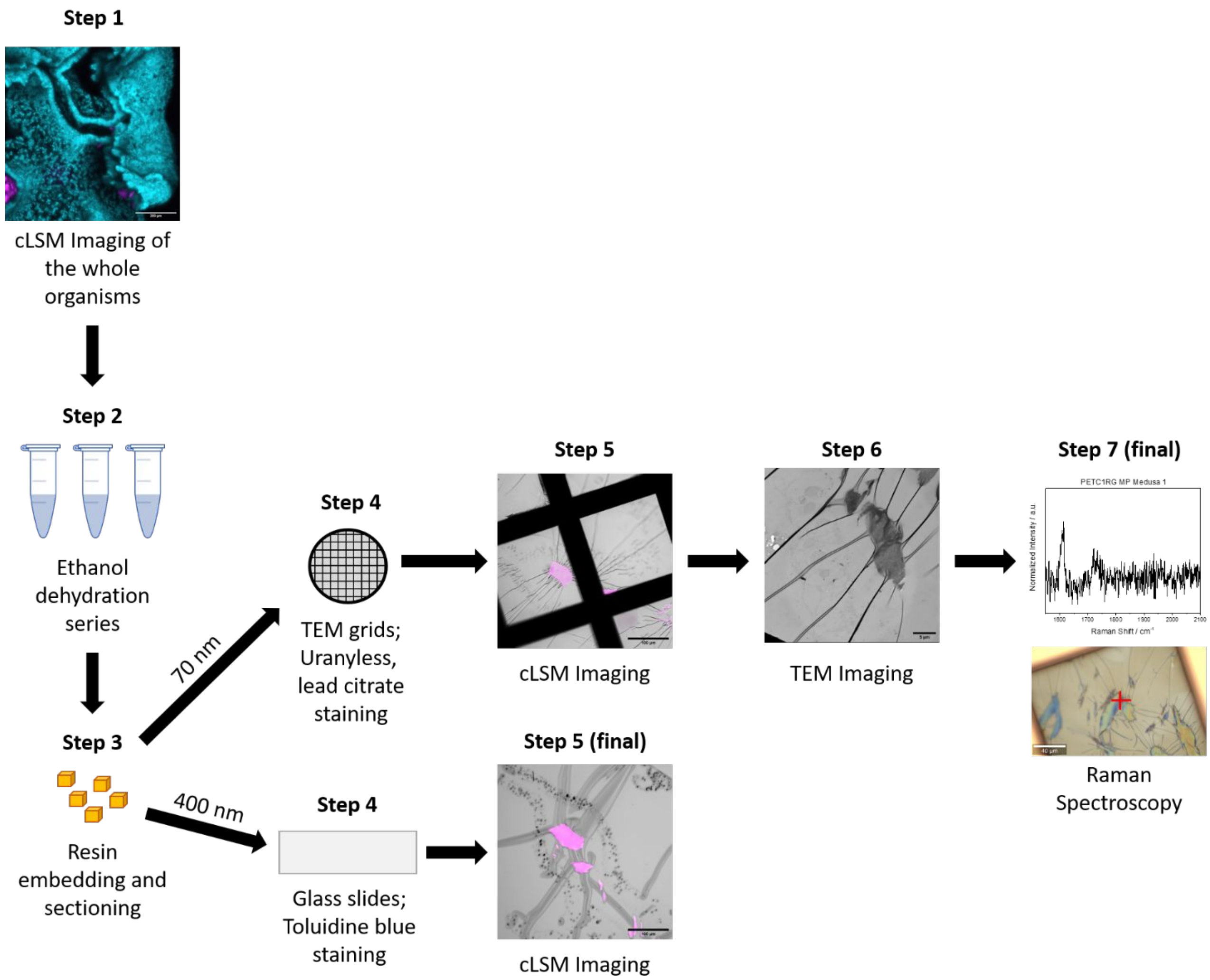

Scheme S1: A stepwise overview of the workflow for samples after MP exposure and chemical fixation.

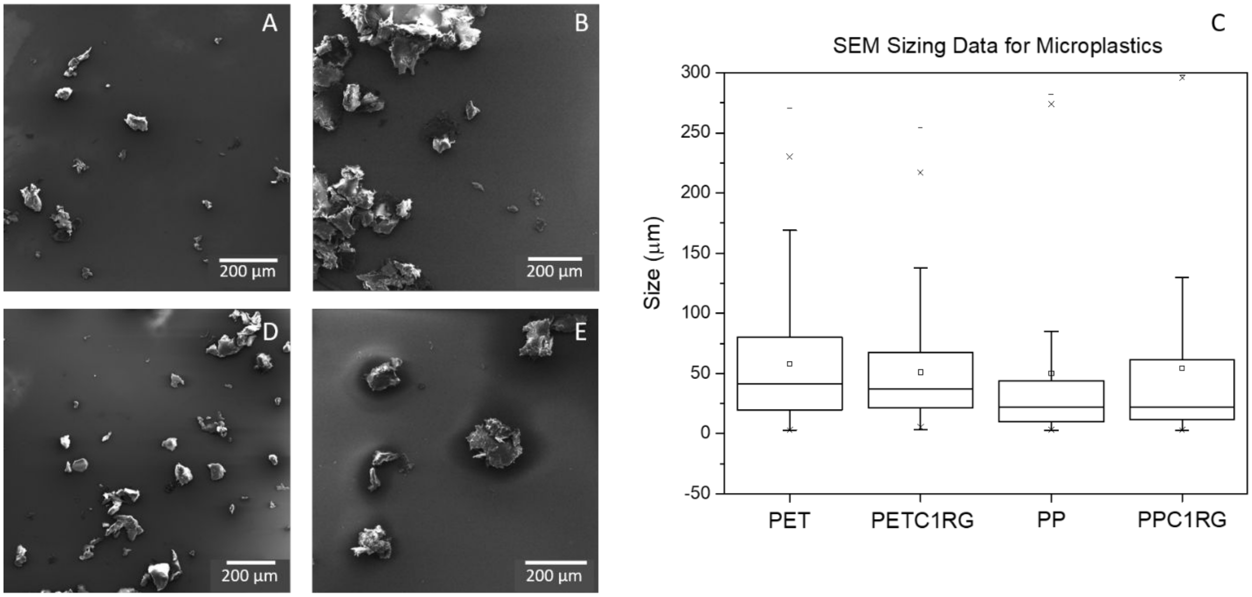

Figure S1: Representative scanning electron microscopy (SEM) images of each milled microplastic particle (MP) stock and a box plot of the corresponding particle size distribution data obtained. Within the box plot, boxes represent first to third quartile data, with the mean value indicated by the small, open square, the median value indicated by a central line, and whiskers used to indicate the minimum (lower) or maximum (upper) value measured. A) 1,4-bis(α-cyano-4-methoxystyryl)-2,5-dimethoxybenzene labelled poly(ethylene terephthalate) (PETC1RG) MPs. B) Labelled polypropylene (PPC1RG) MPs, C) Box plot of sizing data. D) Unlabeled PET MPs. E) Unlabeled PP MPs.

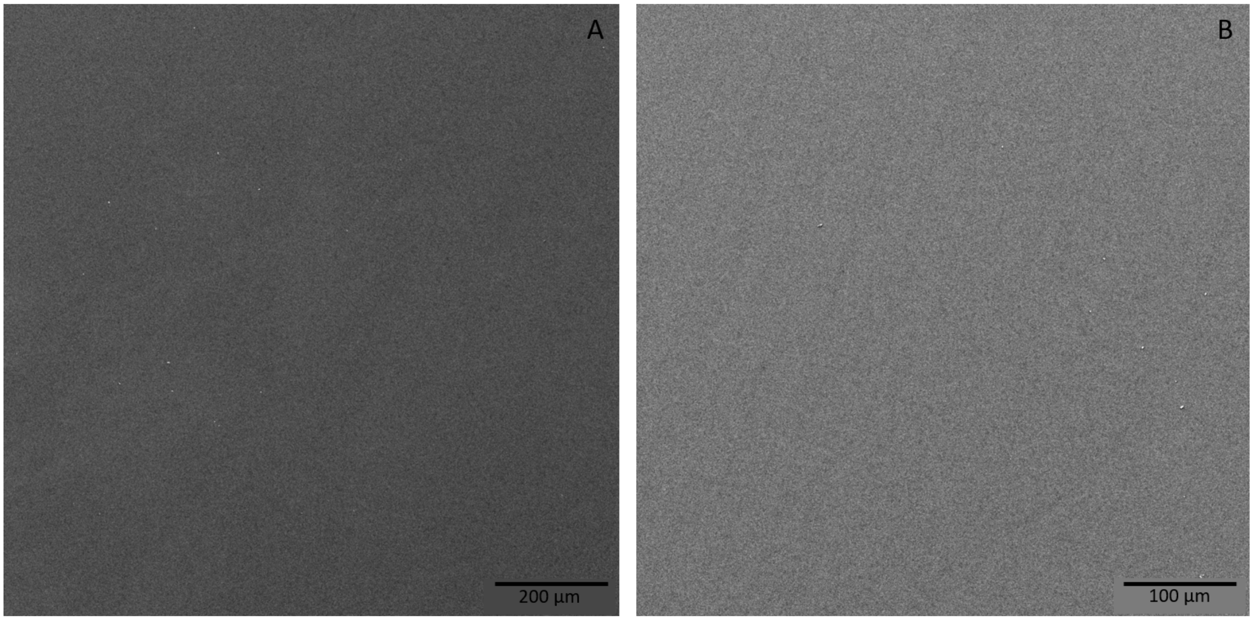

Figure S2: SEM images of the procedural blank samples from MP sizing experiments at two different magnifications; A) the same magnification used for MP sizing and B) a higher magnification.

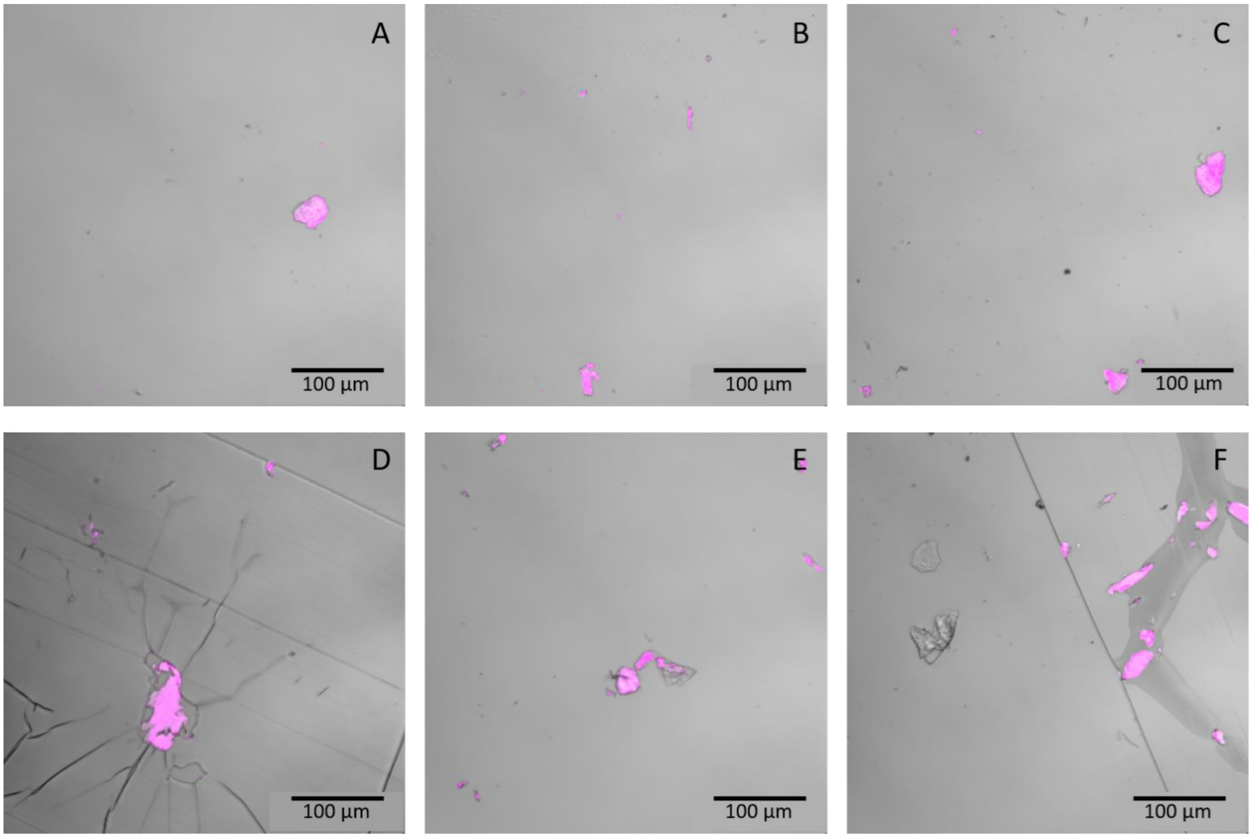

Figure S3: Confocal laser scanning microscopy (cLSM) images from the resin-embedded MP sample sections placed on glass slides. These sections were used to confirm the MP fluorescence was not lost during sample processing and to optimize the section thickness. The fluorescence of C1RG labelled MPs was imaged with EGFP excitation/emission filters and is shown in magenta while the bright field imaging was used to visualize the surrounding resin. A) PPC1RG MPs sectioned at 200 µm thickness. B) PPC1RG MPs sectioned at 500 µm thickness. C) PPC1RG MPs sectioned at 800 µm thickness. D) PETC1RG MPs sectioned at 200 µm thickness. E) PETC1RG MPs sectioned at 500 µm thickness. F) PETC1RG MPs sectioned at 800 µm thickness.

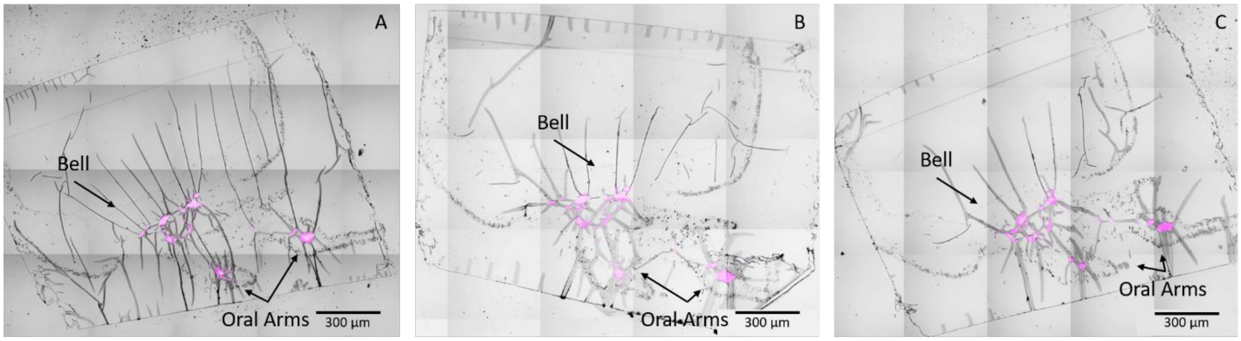

Figure S4: Representative tile scans of sequential 400 nm thick resin-embedded sample sections for PETC1RG MP exposed medusa 1 placed on glass slides. The fluorescence of C1RG labelled MPs was imaged with EGFP excitation/emission filters and is shown in magenta while the bright field imaging was used to visualize toluidine blue stained C. andromeda tissue and endosymbionts. A) The first section. B) The tenth section. C) The twenty-first section.

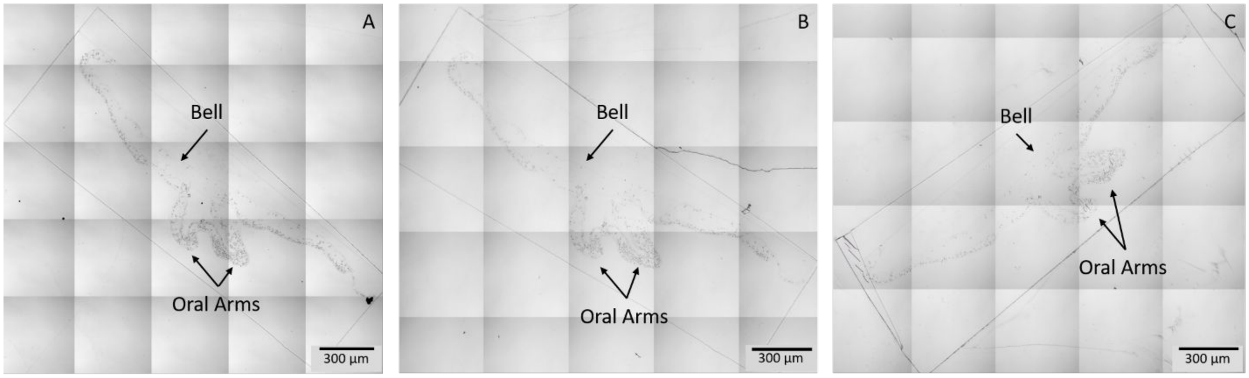

Figure S5: Representative tile scans of sequential 400 nm thick resin-embedded sample sections for an unlabeled PET MP exposed medusa placed on glass slides. The fluorescence of C1RG labelled MPs was checked for with EGFP excitation/emission filters and is shown in magenta while the bright field imaging was used to visualize toluidine blue stained C. andromeda tissue and endosymbionts. A) The first section. B) The fourth section. C) The eighth section.

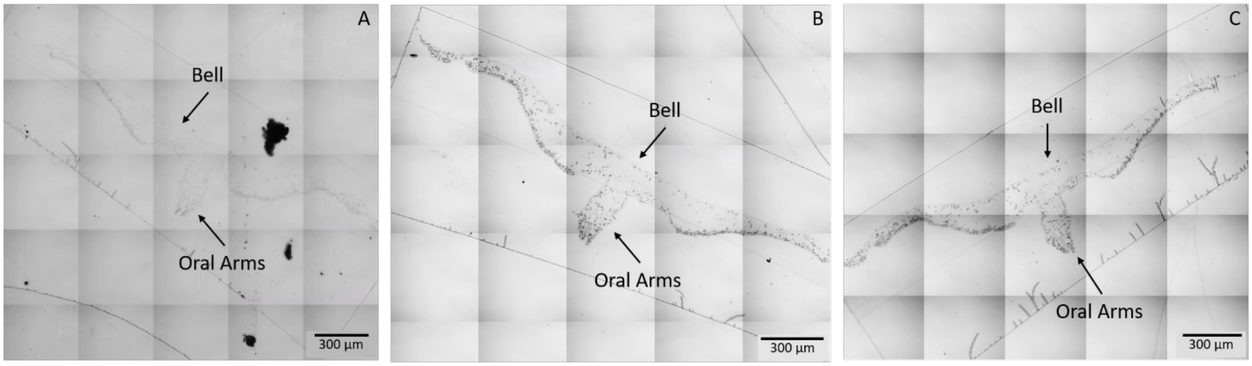

Figure S6: Representative tile scans of sequential 400 nm thick resin-embedded sample sections for a PPC1RG MP exposed medusa placed on glass slides. The fluorescence of C1RG labelled MPs was checked for with EGFP excitation/emission filters and is shown in magenta while the bright field imaging was used to visualize toluidine blue stained C. andromeda tissue and endosymbionts. A) The first section. B) The fourth section. C) The eighth section.

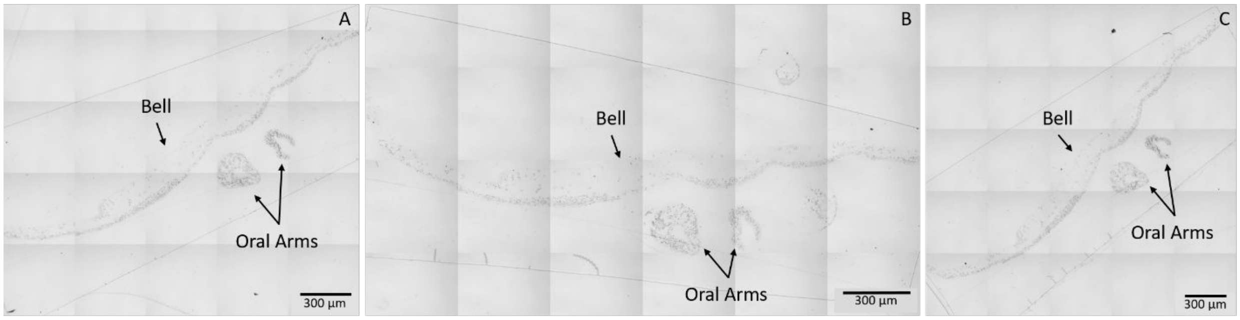

Figure S7: Representative tile scans of sequential 400 nm thick resin-embedded sample sections for an unlabeled PP MP exposed medusa placed on glass slides. The fluorescence of C1RG labelled MPs was checked for with EGFP excitation/emission filters and is shown in magenta while the bright field imaging was used to visualize toluidine blue stained C. andromeda tissue and endosymbionts. A) The second section. B) The fourth section. C) The seventh section.

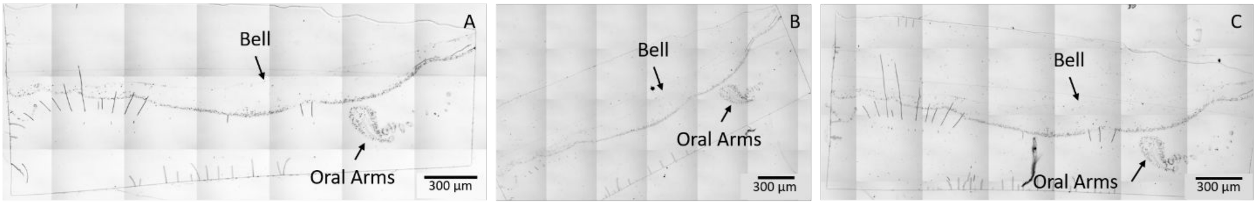

Figure S8: Representative tile scans of sequential 400 nm thick resin-embedded sample sections for a control medusa not exposed to microplastics placed on glass slides. The fluorescence of C1RG labelled MPs was checked for with EGFP excitation/emission filters and is shown in magenta while the bright field imaging was used to visualize toluidine blue stained C. andromeda tissue and endosymbionts. A) The first section. B) The fourth section. C) The eighth section.

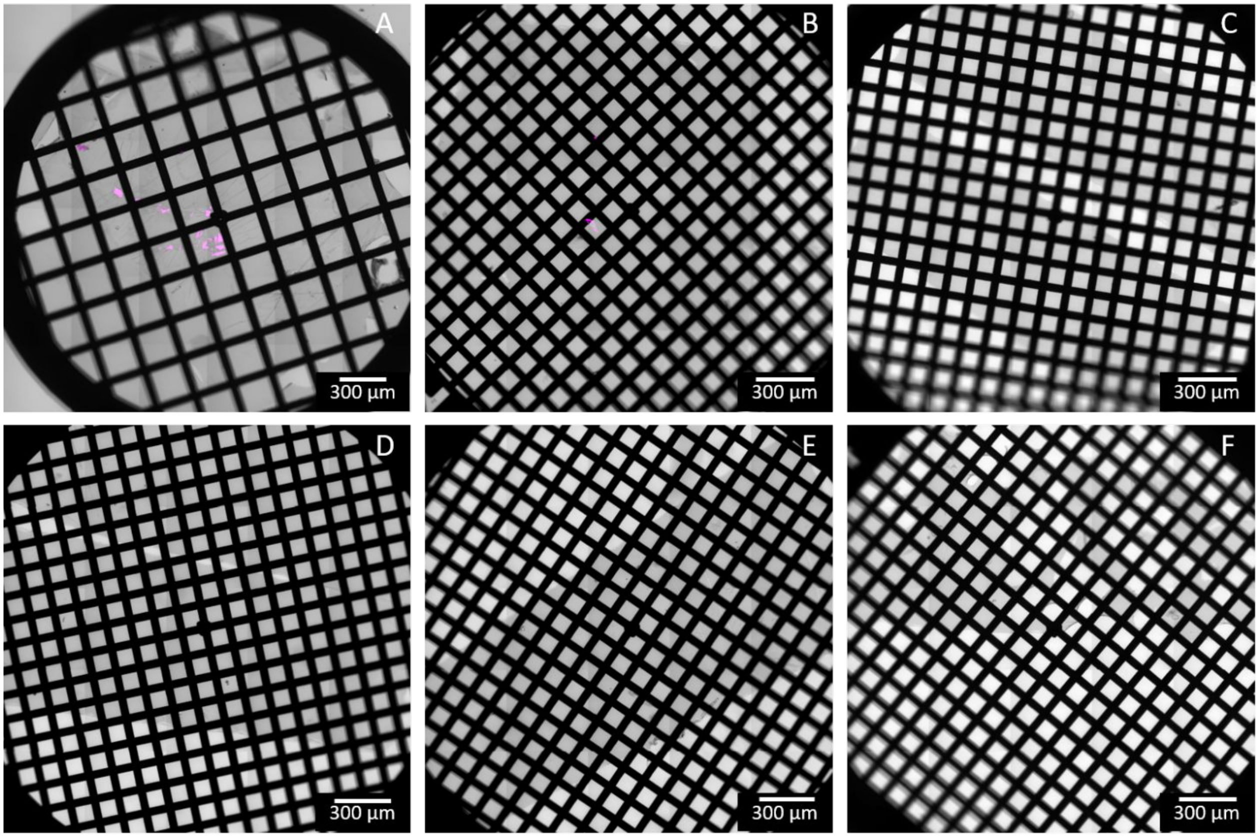

Figure S9: Representative tile scans of 70 nm thick resin-embedded sample sections for each exposure condition placed on transmission electron microscopy (TEM) grids. The fluorescence of

C1RG labelled MPs was imaged with EGFP excitation/emission filters and is shown in magenta while the bright field imaging was used to visualize stained C. andromeda tissue and endosymbionts. A) PETC1RG MP exposed medusa 1. B) PETC1RG MP exposed medusa 2. C) Unlabeled PET MP exposed medusa. D) PPC1RG MP exposed medusa. E) Unlabeled PP MP exposed medusa. F) Control medusa.

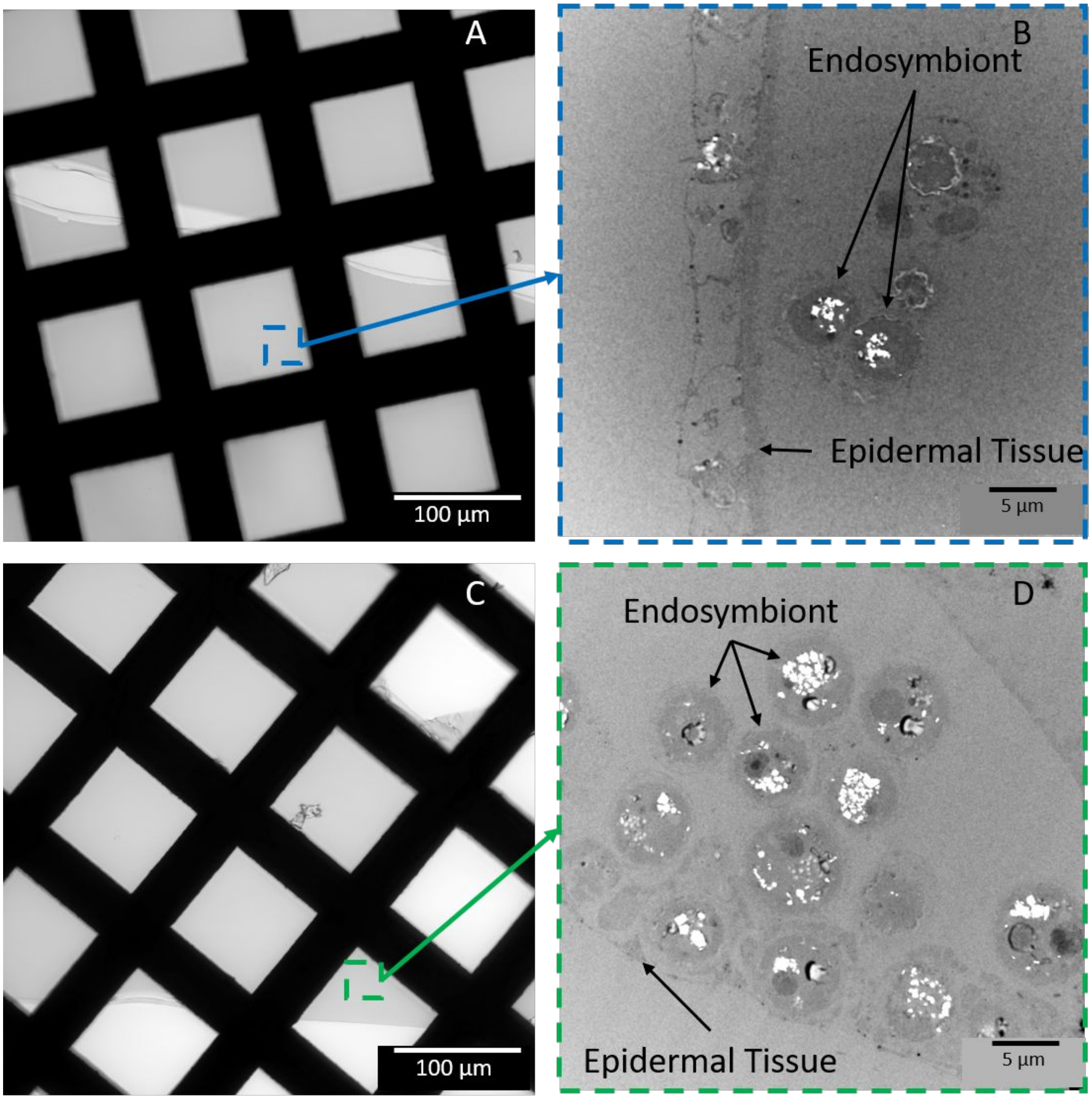

Figure S10: Representative cLSM images from the final 70 nm thick resin-embedded sample sections placed on TEM grids which did not show plastic particle presence. The fluorescence of C1RG labelled MPs was checked for with EGFP excitation/emission filters, shown in magenta, while the bright field imaging was used to visualize toluidine blue stained C. andromeda tissue and endosymbionts. A) A cLSM image of a PPC1RG MP exposed medusa with a blue dashed rectangle and blue arrow highlighting the region of interest shown in TEM image B). C) A cLSM image of a

control medusa which was not exposed to microplastics with a green dashed rectangle and green arrow highlighting the region of interest shown in TEM image D). Representative examples of key features of interest in TEM images have been labelled with arrows for clarity.

Table S1: A summary of important numbers obtained from MP sizing by SEM imaging. Additional values for hydrophobicity (measured through contact angle [1]) and crystalline transition temperatures (T_c; measured through differential scanning calorimetry [1]) are presented to demonstrate there are no major differences in relevant properties of the labeled and unlabeled MPs.

**Microplastic Type**	**Sample Size**	**Minimum Diameter (µm)**	**Maximum Diameter (µm)**	**Average Diameter ± Standard Error (µm)**	**Contact Angle of Water ± Standard Deviation [1]**	**T_c (°C) [1]**
**PETC1RG**	196	3.2	254.4	51.1 ± 3.2	81 ± 3°	205
**Unlabeled PET**	176	2.8	270.9	57.9 ± 3.9	83 ± 5°	185
**PPC1RG**	125	2.5	298.1	48.5 ± 6.4	91 ± 4°	116
**Unlabeled PP**	121	2.4	282.0	50.1 ± 6.3	94 ± 4°	116

Table S2: A list of exact exposure masses for each C. andromeda juvenile medusa used in the study. The exposures were conducted over the course of 6 h in a 12-well plate. Per well one medusa was

present in a final exposure volume of 2.52 mL. The use of 'B' within a listed sample name indicates it was a blank control which was not exposed to MPs.

Sample	Exposure Type	Plastic Mass (mg)
**1**	PET	0.377
**2**	PET	0.333
**3**	PET	0.293
**4**	PP	0.315
**5**	PP	0.358
**6**	PP	0.386
**7**	PPC1RG	0.329
**8**	PPC1RG	0.344
**9**	PPC1RG	0.382
**10**	PETC1RG	0.358
**11**	PETC1RG	0.312
**12**	PETC1RG	0.380
**B1**	Blank Control	_

**B2**	Blank Control	_
**B3**	Blank Control	_
**B4**	Blank Control	_

Table S3: A list of relevant measurement parameters for all Raman spectra shown within the main manuscript and the SI. All measurements were acquired using a 633 nm laser.

Sample	Accumulations	Accumulation Time (s)	Laser Power (mW)	Measurement Grating (gr/mm)
**PET MP Control on Glass**	300	0.5	5	300
**PP MP Control on Glass**	300	0.5	5	300
**Procedural Blank Medusa TEM Section**	750	0.5	1	300
**PET MP Exposed Medusa TEM Section**	750	0.5	1	300

**PETC1RG MP Exposed Medusa 1 TEM Section**	1,000	0.5	1	300
**PETC1RG MP Exposed Medusa 2 TEM Section**	1,000	0.5	1	300
**PPC1RG MP Exposed Medusa TEM Section**	750	0.5	1	300

REFERENCES

1. Caldwell, J.; Lehner, R.; Balog, S.; Rhême, C.; Gao, X.; Septiadi, D.; Weder, C.; Fink, A. S.; Rothen-Rutishauser, B., Fluorescent Plastic Nanoparticles to Track their Interaction and Fate in Physiological Environments. *Environmental Science: Nano* **2021,** *8*, 502-513.
